# Case Series of First Microinvasive Fully Endoscopic Use of a New Mitral Prosthesis

**DOI:** 10.3390/jcm13154358

**Published:** 2024-07-25

**Authors:** Jacqueline Kruse, Miriam Silaschi, Kristina Russu, Alina Kirschen, Farhad Bakhtiary

**Affiliations:** Department of Cardiac Surgery, Heart Center Bonn, University Hospital Bonn, 53127 Bonn, Germany; miriam.silaschi@ukbonn.de (M.S.); kristina.russu@ukbonn.de (K.R.); alina.kirschen@ukbonn.de (A.K.); farhad.bakhtiary@ukbonn.de (F.B.)

**Keywords:** mitral valve replacement, microinvasive valve surgery, biological valves

## Abstract

The use of bioprostheses is increasing in younger patients, but it is associated with the risk of later valve deterioration, especially in the mitral position. A new bioprosthesis for mitral valve replacement offers possible longer-term durability and improved hemodynamics. **Objectives**: Here, we report the implantation of the novel Edwards MITRIS RESILIA mitral valve (Edwards Lifesciences Inc., Irvine, CA, USA) through microinvasive fully endoscopic access as an innovative surgical approach based on a series of twelve patients. **Methods**: Contrast-based ECG gated CT was preoperatively performed in all patients to determine the intravascular calcifications and vascular parameters, as well as to assess noticeable problems during the operation. CT software for cardiac interventions (3Mensio Medical Imaging BV) was used to simulate surgical prostheses digitally inside the native annulus. With this, a digital LVOT and neo LVOT was created, and the difference between the valve prostheses was measured. Implantation of the MITRIS RESILIA valve was performed in 12 patients according to the instructions for use through microinvasive access in a fully endoscopic fashion using 3D visualization. **Results**: The mean patient age was 56.50 years, and 7/12 (58.33%) were redo procedures. All patients survived the first 30 days after the procedure, the mean aortic cross-clamp time was 40.17 ± 13.72 min. and mean postoperative transvalvular gradient was 4.45 ± 1.74 mmHg. The neo LVOT in the CT-based simulation was measured with an average area of 414.98 ± 88.69 mm^2^. The average difference between the LVOT and neo LVOT area was 65.35 ± 34.99 mm^2^. There was no case of paravalvular leakage or obstruction of the left ventricular outflow tract. **Conclusions**: The novel MITRIS RESILIA valve is a promising new bioprosthesis for mitral valve replacement that offers improved features as compared to other prostheses. The ease of implantation is increased by this prosthesis by the improved pliability of the sewing cuff and the inward folding of the struts, which was confirmed by short operative times in our series.

## 1. Introduction

Minimally invasive mitral valve surgery (MIMVS) has evolved significantly over the past few decades, driven by advancements in technology and surgical techniques. The minimally invasive approach involves a small incision made in a right mini-thoracotomy, which reduces surgical trauma, promotes quicker recovery, and lowers the risk of complications like surgical wound infections and nosocomial infections [1].

Studies have shown that MIMVS is not only feasible and safe but also effective in treating complex mitral valve diseases, including infective endocarditis and mitral regurgitation, with comparable or better outcomes than conventional sternotomy [2]. The use of high-definition 3D visualization and specialized instruments allows for precise and accurate repairs, even in complex cases [3]. Additionally, the development of comprehensive preoperative imaging and simulation techniques has further enhanced the safety and success rates of these procedures, allowing for tailored surgical strategies based on individual patient characteristics. 

In patients with mitral valve disease, repair is the preferred route of treatment, as short- and long-term outcomes have been reported to be superior. However, if a durable repair is not feasible, replacement should be performed, preferably with preservation of the subvalvular apparatus of the mitral valve. Younger patients with mitral valve disease face various lifestyle restrictions depending on the type of mitral valve intervention they undergo [4]. Regular physical activity is encouraged as it reduces all-cause mortality rates, but it is crucial to balance the benefits with the potential risks of triggering major cardiac events due to changes in hemodynamic balance during exercise [5]. For patients with mitral valve replacement, the choice between mechanical- and bioprostheses significantly impacts the lifestyle and lifetime risk of stroke and bleeding, as well as the risk for structural valve deterioration. Mechanical mitral valves require lifelong oral anticoagulation, which precludes successful pregnancy and necessitates careful management and patient compliance to avoid thromboembolic complications [6,7]. Tissue valves, while preferable for women planning pregnancy, due to their lower anticoagulation requirements and association with a low overall risk of stroke and bleeding, tend to degenerate faster, often necessitating repeat interventions [8]. 

However, in younger patients (<60 years), guidelines still favor the use of mechanical valves, despite these disadvantages [9]. In addition, the possibility of later catheter-based treatment offers additional strategies to avoid later re-operation in patients with biological prostheses [10].

However, with various bioprostheses available, the long-term durability is still limited, and even with catheter-based treatments available, lifetime management in very young patients remains problematic. The MITRIS RESILIA valve (EDWARDS Lifesciences Inc., Irvine, CA, USA) is designed to increase the durability of bioprostheses in the mitral position further and to reduce the peri-operative complications with an increased ease of implantation. RESILIA tissue is a bovine pericardial tissue, which has been treated to attenuate the free aldehydes to ensure a longer durability. 

The long-term durability of prosthetic valves made with RESILIA tissue has been demonstrated by an extensive set of research that includes preclinical investigations, clinical trials, and registry data. With a 1.0% incidence in propensity-matched cohorts over a five-year period, the COMMENCE trial revealed that RESILIA tissue valves had significantly lower rates of hemodynamic valve deterioration (HVD) due to structural valve deterioration (SVD) than did modern AVR cohorts [11]. With a mean gradient of 13.3 mmHg at two years and a two-year freedom from all-cause mortality of 97.4% for isolated AVR and 95.7% for all patients, the RES-ITA Registry, which comprised around 1000 patients, encountered excellent early and mid-term results [12]. Furthermore, a five-year Polish research study with significant improvements in patient functional status and hemodynamic performance revealed no reports of SVD [13,14]. The INSPIRIS RESILIA Durability Registry (INDURE) also reported good safety and hemodynamic performance at one year, with no cases of stage 3 SVD and a mean aortic pressure gradient of 12.6 mmHg [15,16]. Accelerated wear testing (AWT) indicates that the INSPIRIS RESILIA valve maintained outstanding hemodynamic performance after the equivalent of 25 years of usage, exceeding 1 billion cycles of simulated wear. Preclinical studies further confirm these findings [17]. Moreover, economic evaluations suggest that RESILIA tissue valves offer substantial long-term cost savings compared to mechanical valves, primarily due to the reduced calcification and SVD [18]. Collectively, these studies provide compelling evidence that RESILIA tissue in prosthetic valves offers prolonged durability, excellent hemodynamic performance, and significant clinical benefits over both short- and long-term follow-up periods.

The aortic valve prostheses already in routine use show a 97% freedom from valve degeneration after 7 years. Improved durability can therefore also be expected for the mitral valve.

Furthermore, it is low profile, with the lowest effective anterior stents compared to commonly available mitral valve prostheses and an asymmetric design of the sewing cuff which tilts the posterior strut away from the LV wall to prevent left ventricular outflow tract obstruction [19]. A fabric skirt and ventricular anchors are also included in this design to provide a snug fit and efficient sealing along the mitral valve annulus [4].

Here, we report the surgical technique of implanting the MITRIS RESILIA through fully endoscopic access using 3D visualization in 11 patients. This was accompanied by de-calcification of the mitral annulus by ultrasonic aspirator Sonopet iQ (Stryker Osteonics SA, Biberist, Switzerland) in one patient [20]. In another patient, we implanted the MITRIS RESILIA valve in a tricuspid position. 

## 2. Materials and Methods

For pre-operative planning, at our department, we perform a contrast-based ECG gated CT as a standard of care to be able to anticipate possible intraoperative problems. It is performed from the supra-aortic vessels to the caudal area of the inguinal vessels. This serves to determine intravascular calcifications and vascular parameters, as well as to recognize the position of the heart in the thorax and to be able to assess any problems during the operation.

We then use dedicated CT software for cardiac interventions (3Mensio Medical Imaging BV, Utrecht, The Netherlands) and simulate surgical prostheses digitally inside the native annulus. We are thus able to measure the appropriate valve sizes beforehand, measure the distance of the mitral annulus to the circumflex artery, quantify the annular calcifications, measure the length of the anterior mitral leaflet, create a digital neo LVOT and compare the values between different prostheses. In microinvasive endoscopic surgery, direct measurement is cumbersome; therefore, it eases the procedural steps to measure the annular sizes digitally beforehand. 

The procedural steps of our microinvasive fully endoscopic valve replacement procedure are as follows:

During general anesthesia, cardiopulmonary bypass is established via femoral or even axillary vessels in case of aorto–iliac calcifications or aortal kinking. An additional venous neck cannula is placed through the internal jugular vein. The procedure is performed under transesophageal guidance and full heparinization of the patient. We perform the procedure in normothermia; however, if need be, mild hypothermia may be applied.

Mini-thoracotomy is performed through the third or fourth intercostal space and the site is exposed using a soft tissue retractor. A camera port is inserted for 3D endoscopy, as well as the transthoracic aortic clamp. The pericardium is opened above the phrenic nerve.

Separation of the aorta from the pulmonary artery, release of the adhesions on the aorta, and insertion of the Chitwood aortic clamp via another stab incision in the thorax through the second intercostal space is then performed. Next, we perform cross clamping of the aorta and administration of Brettschneider crystalloid cardioplegia solution via the aortic root. The left atrium is opened through the interatrial grove and exposed with a retractor after cross clamping the aorta and the induction of cardioplegic arrest. Then, the mitral valve is exposed using a dedicated retractor and excised with preservation of the subvalvular apparatus if possible. At a minimum, the posterior leaflet with its subvalvular structures should be preserved. Subsequently, if needed, we use an ultrasonic aspirator to decalcify the annulus, thus enabling the proper placement of sutures as well as prosthesis function.

**Figure 1:**
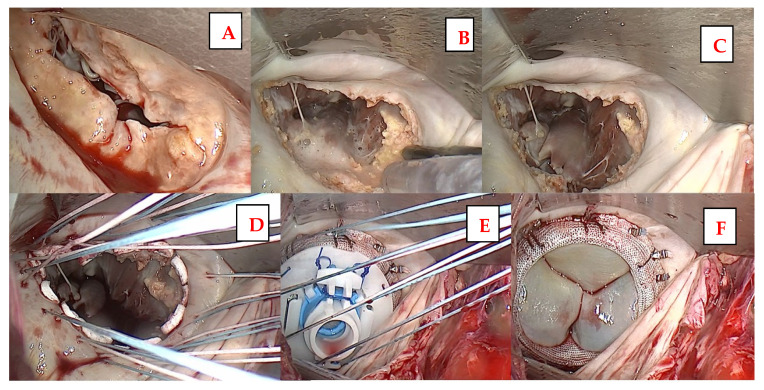
Microinvasive fully endoscopic implantation of the MITRIS RESILIA mitral valve prosthesis. (**A**): Native mitral valve with a combined pathology (regurgitation and stenosis), (**B**): ultrasonic decalcification of the mitral annulus after excision of the native leaflets, (**C**): result after decalcification, (**D**): insertion of the valve sutures, (**E**): implantation of the MITRIS RESILIA mitral valve, (**F**): final result of the implanted MITRIS RESILIA mitral valve.

MITRIS RESILIA is implanted in a standard fashion in the mitral valve position (see Supplementary Video S1, see Figure 1). There is ease of implantation through the microinvasive access due to the flexible stent, which can be temporarily adjusted to a 55-degree angle. After implantation and waterprobe, the left atrium is closed using double-layered continuous sutures. Then, further aortic valve replacement may be performed if needed (case 7). In such a case, aortotomy is performed, excision of the aortic valve, decalcification and sizing of the annulus, and subsequent implantation of a biological prosthesis.

In the case of the implantation of the MITRIS RESILIA valve in the tricuspid position (case 10), the operation was performed on a beating heart. Total cardiopulmonary bypass was performed by occluding the superior and inferior vena cava using crocodile clips and two venous cannulas (neck and groin) for drainage. 

After completion of valve implantation, the aortic cross clamp is removed and the heart de-aired; then, weaning from cardiopulmonary bypass and decannulation is performed using the MANTA^®^ Vascular Closure Device (Teleflex Incorporated, Wayne, PA, USA) in case the femoral vessels were cannulated. Details of the first 11 patients treated with the MITRIS RESILIA valve can be seen in Table 1. 

## 3. Results

Patients who received a new Edwards MITRIS RESILIA valve at our center were on average 56.5 ± 18.7 years old. In total, 58.33% (7/12) of the patients received a redo operation. The average time of cardiopulmonary bypass was 66.83 ± 14.27 min, and the cross-clamp time was on average 40.17 ± 13.72 min. A total of 66.67% (8/12) of the patients were extubated on the operating table. The average size of the valve was 30.17 ± 1.28 mm. In one patient, prior to implantation, the ultrasonic aspirator Sonopet iQ for decalcification of the mitral annulus was used. In one patient, the procedure was a double-valve procedure (mitral valve prosthesis combined with aortic valve replacement). The average length of the hospital stay was 10.42 ± 6.1 days (some patients had a prolonged hospital stay due to the presence of endocarditis, which needed longer antibiotic therapy), and the mean ICU stay was 2.92 ± 1.74 days.

The postoperative result of the valve implantation showed no paravalvular leakage in any of the 12 patients. The mean gradient across the mitral valve prosthesis was 4.45 ± 1.74 mmHg. At 90 days follow-up, all patients were alive. All patients (11/11) were in NYHA class I-II, and no patient was re-hospitalized for cardiac reasons. The results of the individual patients are listed in Table 1. 

The neo LVOT in the CT-based simulation was measured with an average area of 414.98 ± 88.69 mm^2^. The average difference between the LVOT and the neo LVOT area was 65.35 ± 34.99 mm^2^ on average; its reduction in area was numerically less pronounced than compared to another valve prosthesis (Epic: 68.69 ± 32.71 mm^2^ (Epic, Abbott Cardiovascular Inc., St. Paul, MN, USA)).

## 4. Discussion

Bioprostheses are increasingly used in the mitral position in younger patients, despite the known disadvantages of the routinely available prostheses. The option of later catheter-based treatment and the awareness of the increased lifetime risk of stroke with mechanical valves has pushed this trend further. 

The MITRIS RESILIA may likely overcome some of these disadvantages, with its improved durability, potentially less risk for LVOT obstruction, and ease of implantation, thus facilitating minimally or microinvasive approaches in younger patients. 

The results of our case series reflect some of these features, as the cross-clamp times were relatively short (<1 h), and the mean gradients were satisfactory. No patient had paravalvular leakage or LVOT obstruction. No patient was converted to sternotomy. The ICU stay postoperatively was quite short; only one patient was transferred back to the ICU because of respiratory insufficiency. The postoperative stay at the hospital was longest for the patient with respiratory insufficiency and for the patient with a preoperative diagnosed endocarditis and the associated postoperative antibiotic therapy. Despite these positive short-term outcomes, long-term outcomes need to be reported in future in order to assess the real-world durability. There are already some registries collecting further outcome data for the Resilia tissue, as for example the Durability Registry (INDURE), which reported good safety and hemodynamic performance at one year, with no cases of stage 3 SVD.

After seven years, the Inspiris RESILIA aortic valve prosthesis currently in common use exhibits a 97% freedom from valve degeneration. As a result, increased durability for the mitral valve is also anticipated. Accelerated wear testing (AWT) of the Inspiris RESILIA aortic valve demonstrated a durability equivalent to 25 years, meeting ISO standards and showing only minimal changes in hemodynamic performance [14,15].

Several studies have compared the longevity of prosthetic valves with and without RESILIA tissue, demonstrating significant differences in durability and performance. The COMMENCE trial compared structural valve deterioration (SVD)-related hemodynamic valve deterioration (HVD) of RESILIA tissue valves to those from the PARTNER 2A trial, showing reduced SVD-related HVD in the RESILIA group (1.8% vs. 3.5%) over five years [11]. 

The MITRIS RESILIA mitral valve prosthesis offers several key benefits in mitral valve replacement procedures. It provides a unique profile and innovative option for patients at risk of left ventricular outflow tract obstruction, making it a valuable choice for individuals in need of mitral valve replacement at a young age [4]. We assume the thicker cuff is a feature lowering the risk of paravalvular leakage. 

The special feature of the MITRIS RSILIA valve, apart from its contours of the ring which fit better into the geometric shape of the annulus and its flexible stent for an easier way of implantation through the microinvasive cut, is that the height of the prosthesis is less than 7 mm, which may be associated with a lower risk of LVOT obstruction. 

When a mitral valve is replaced, especially in the presence of mitral annular calcification, the new valve can displace the anterior mitral leaflet towards the LVOT, creating a new obstruction (NEO-LVOT). The design of the MITRIS RESILIA mitral valve is intended to significantly reduce the risk of LVOT obstruction. The larger remaining area of the NEO-LVOT also results in improved hemodynamics under stress, which is particularly positive in young patients. In our series, we did not measure the LVOT flow velocity in postoperative echocardiography; however, we measured the neo LVOT area in the digital prosthesis simulation and found a numerically less pronounced reduction than in another commercially available prosthesis. However, to account for the significance, further studies with more patients are needed. This study was not powered to sufficiently compare the LVOT geometry. We, as a center of excellence in heart valve surgery, strongly recommend pre-operative CT planning and digital simulation. Pre-operative CT has been shown to reduce peri-operative complications with regard to the cannulation strategy (REF), and we have further evolved CT planning by digital prosthesis simulation strategies, which we strongly recommend in microinvasive surgery [21]. A meta-analysis reported a superior safety profile with pre-operative CT planning already in 2018, however, this has not been adopted by every center performing minimally invasive valve surgery so far. The stroke rate was significantly reduced (CT-group: 1.5% vs. Non-CT: 2.2%, *p* = 0.03), and the need for dialysis was also significantly reduced (0.8% vs. 2.3% in the Non-CT group, *p* = 0.02) [22].

The Resilia tissue tends to calcify less, a primary cause of structural valve deterioration, leading to promising long-term durability and cost savings compared to mechanical valves [8]. 

Different materials are used in the construction of the Mitris prosthesis to ensure durability and functionality, as already seen in the INSPIRIS RESILIA aortic prosthesis. It is known that the durability of the mitral valve is significantly lower than that of the aortic valve. Studies have shown that these new generation aortic bioprostheses with Resilia tissue demonstrate good early and mid-term outcomes, versatility of use, and excellent hemodynamic performance, with low rates of complications such as paravalvular leaks and prosthesis dysfunction [4]. However, there are no very-long-term data comparable to the Hancock (Medtronic Inc., Minneapolis, MN, USA) or Perimount prosthesis (EDWARDS Lifesciences Inc., Irvine, CA, USA). Future data will assess whether the promise of the improved durability of RESILIA tissue holds true beyond 10 years after implantation. In addition, its durability in patients on chronic dialysis must be assessed, since this is another Achilles heel of using bioprostheses. The further development of the mitral valve with RESILIA tissue may significantly extend the durability of the valve and provide a further option for young patients compared to the use of mechanical valves. 

## 5. Conclusions

Implantation of the novel Edwards MITRIS RESILIA valve through microinvasive fully endoscopic access is a promising concept in the treatment of patients with mitral valve disease not feasible for repair. It offers several key benefits, including improved hemodynamic performance and durability. The ease of implantation is increased by this prosthesis through the improved pliability of the sewing cuff and the inward folding of the struts. This is reflected by short aortic cross-clamp times. The implantation through microinvasive access is a safe procedure and could be performed in a regular manner.

Overall, the MITRIS RESILIA mitral valve combines unique features with clinical efficacy, making it a valuable option in the landscape of mitral valve prosthesis.

## 6. Limitations

This study is a retrospective study with no randomization comprising only 11 cases. Due to the fact that the heart valve has not been implanted for a very long time, we cannot make any statement about the long-term results. The hemodynamics across the MITRIS RESILIA valve prothesis were assessed using the mean gradient, which depends on various factors (such as preload, afterload, filling pressures, heart rate, recent exertion, and further criteria). No follow-up values for the mean gradient were analyzed. 

However, this is the first microinvasive fully endoscopic experience of this new tissue valve. 

## Figures and Tables

**Table 1:** Patient characteristics and outcomes.

	#1	#2	#3	#4	#5	#6	#7	#8	#9	#10	#11	#12
Age	57	69	37	66	52	71	24	77	69	19	66	71
REDO procedure	√	X	√	X	X	√	√	X	√	√	√	√
MAC	√	X	X	X	X	X	√	X	√	X	X	X
Type of MV dysfunction	Mixed	Regurgitation (M. Barlow)	Regurgitation + LVOT obstruction by SAM (M. Barlow)	Regurgitation	Mixed	Endocarditis	Stenosis (shone complex)	Regurgitation	Regurgitation	Tricuspid valve regurgitation	Regurgitation	Regurgitation
Cannulation	Femoral	Femoral	Femoral	Femoral	Femoral	Femoral	Femoral	Axillary	Axillary	Femoral	Femoral	Femoral
Aortic cross clamp time (min.)	54	29	62	23	45	35	16	61	38	47	33	39
Cardiopulmonary bypass time (min.)	78	46	83	41	83	56	75	82	66	69	51	72
Additional procedures	Ultrasonic decalcification	X	PFO closing	X	Atriclip + Cryoablation	X	Aortic valve replacement	Atriclip + Cryoablation	X	X	X	X
Conversion to sternotomy	X	X	X	X	X	X	X	X	X	X	X	X
Valve size used (mm)	29	29	31	29	33	31	29	31	29	31	29	31
Extubated on operating table	X	√	√	X	√	X	√	√	√	√	√	√
Number of nights in ICU	2	1	2	11	1	3	1	4	1	5	1	3
Postoperative complications	AV-Block III	X	Atrial fibrillation	Respiratory insufficiency	X	X	X	X	Delirium	Re-thoracotomy for bleeding	X	X
Mean mitral gradient at discharge (mmHg)	8.8	4.6	4.0	2.8	5.6	3.2	4.9	5.0	2.3	3.7	2.7	5
Paravalvular mitral leak	X	X	X	X	X	X	X	X	X	X	X	X
Length of postoperative hospital stay (days)	5	4	10	20	8	20	8	7	19	15	3	6

MAC: mitral annular calcification, MV: mitral valve, ICU: intensive care unit, AV: aortic valve.

## Data Availability

The data that support the findings of this study are available from the corresponding author, Jacqueline Kruse, upon reasonable request.

## Supplementary Materials

The following supporting information can be downloaded at: https://www.mdpi.com/article/10.3390/jcm13154358/s1, Video S1: Implantation of a microinvasive fully endoscopic MITRIS RESILIA mitral valve prosthesis.
